# Fluidized Bed Chemical Vapor Deposition on Hard Carbon Powders to Produce Composite Energy Materials

**DOI:** 10.1021/acsomega.4c00297

**Published:** 2024-03-07

**Authors:** Marianna Casavola, Lindsay-Marie Armstrong, Zening Zhu, Daniela Ledwoch, Matthew McConnell, Paul Frampton, Peter Curran, Gillian Reid, Andrew L. Hector

**Affiliations:** † School of Chemistry, University of Southampton, Southampton SO17 1BJ, United Kingdom; ‡ School of Engineering, University of Southampton, Southampton SO17 1BJ, United Kingdom; § Deregallera Ltd, Unit 2, De Clare Court, Pontygwindy Industrial Estate, Caerphilly CF83 3HU, U.K.; ∥ School of Mechanical and Design Engineering, University of Portsmouth, Anglesea Building, Portsmouth PO1 3DJ, U.K.

## Abstract

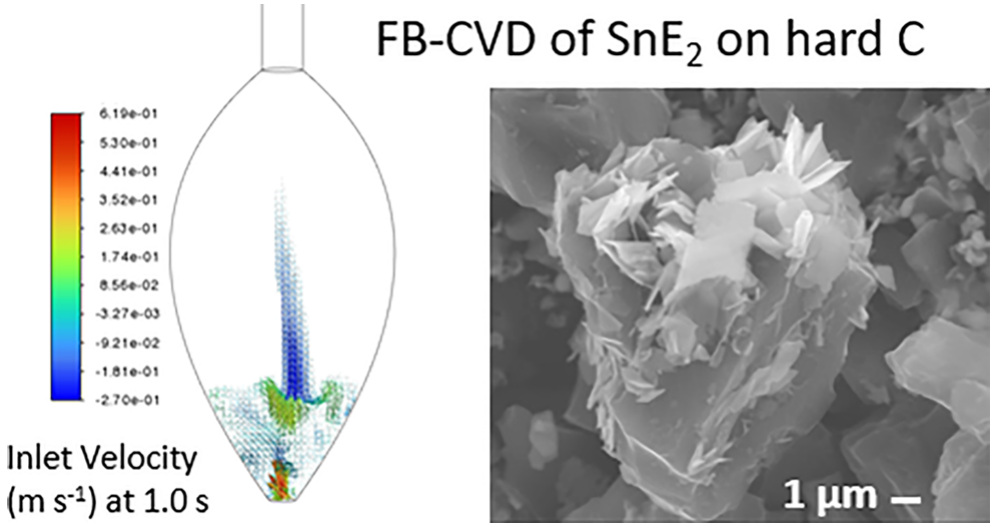

Herein, we report a general route for the uniform coating of hard carbon (HC) powders via fluidized bed chemical vapor deposition. Carbon-based fine powders are excellent substrate materials for many catalytic and electrochemical applications but intrinsically difficult to fluidize and prone to elutriation. The reactor was designed to achieve as much retention of powders as possible, supported by a computational fluid dynamics study to assess the hydrodynamic behavior for varying gaseous flow rates. Solutions of the tin seleno- and thio-ether complexes [SnCl_4_{^*n*^BuSe(CH_2_)_3_Se^*n*^Bu}] and [SnCl_4_{^*n*^BuS(CH_2_)_3_S^*n*^Bu}] were used as single source precursors and injected at high temperature into a fluidized bed of HC powders under nitrogen flow. The method allowed for the synthesis of HC-SnS_*x*_–SnSe_2_ composites at the gram scale with potential applications in electrocatalysis and sodium-ion battery anodes.

## Introduction

Composite materials are fundamental for many applications in energy storage, energy conversion, and catalysis. The combination of cheap, environmentally friendly carbon-based materials and inorganic materials with controllable catalytic, electronic, and optical properties is a winning strategy to fabricate versatile materials for energy applications.^1,2^ Carbon materials are common substrates for the fabrication of heterogeneous catalysts, as by surface modification they can stabilize nanoparticles and achieve tunable catalyst–support interactions.^3^ Carbon-supported semiconductors are key materials for advanced photoelectrocatalysis, with important implications in the removal of pollutants from water and for energy conversion applications.^4^ In these composites, the formation of a heterojunction can greatly enhance electron–hole pair separation and promote electron transfer over recombination mechanisms. Carbon composites are also of great interest for battery electrodes, as highly conductive supports with a large surface-to-volume ratio on which other active materials can be deposited, while the carbon itself can also provide capacity.

In this study, we focused on the synthesis of tin chalcogenide (SnE_*x*_) composites with HC powders, which could be extended to several material combinations. Tin chalcogenides (SnE_*x*_) are semiconductor materials of particular importance for several technological applications in modern electronics. Their layered structure with weak van der Waals interactions between planes makes them structural analogues with graphene. Interestingly, the band gap of the material can be changed by choosing appropriate chalcogens or their combination with tunable band gaps in the 1–2 eV range. On the other hand, weakly coupled layers allow for confined charge, spin, and heat transfer.^5−13^ In this respect, tin chalcogenides hold a high potential for several applications in electronics, such as optoelectronics, thermoelectrics, and phase change devices,^6,10,14−19^ and in photocatalysis and electrocatalysis.^20−23^ In particular, they are a high capacity active material for Na-ion battery anodes.^24−26^ Hence, the deposition of metal dichalcogenides with controlled composition, good substrate/deposit contact, and uniform dispersion on HC powders can be used to exemplify depositions onto carbons to produce functional materials.

The most common synthetic methods for carbon-based materials-inorganic composites are wet-chemical methods, including hydrothermal deposition, incipient wetness impregnation, and coprecipitation, which are limited in the types of materials, which can be deposited and may not provide uniform, well contacted layers on the surface of the particles.^3^ Chemical vapor deposition (CVD) has significant advantages for the deposition of semiconductors, but its use to treat powders, such as carbon materials, is still particularly challenging. CVD can often be used to form coatings with high adherence to the substrate but may not produce uniform coatings on powders. Precursors may not penetrate a layer of substrate particles in a conventional CVD geometry, and the coating uniformity can be affected. An effective way to overcome this limitation is plug flow-CVD, in which the powders are not packed but slightly spread over a small area, allowing for the precursor to be more uniformly adsorbed. This could be an effective method to treat small amounts of powders with several materials and controlled loading, but it is not easily scalable to the treatment of grams of powders. The use of fluidized beds of particles is more scalable and has been widely used in industrial coating and treatment processes. The particles are suspended on a porous plate (distributor) in the reactor bed, and a flow of gas from the bottom of the plate is increased until the particles move as a fluid. In such fluidized regimes, the particle surface areas are exposed during the process and uniform coatings or treatments can be achieved. The ability of particles to be fluidized is described by the Geldart classification, showing that only particles with greater size (tens to hundred μm) and density, thus belonging to the A and B groups, can be stably fluidized.^27−29^ For this reason, FB-CVD has found important industrial applications for, and has been limited to, the treatment of particles such as silica, sand, alumina, stainless steel, metals, and metal carbides, which consist of coarse granules of hundreds of micrometers in diameter and dense material.^30−39^ The fluidization of finer particles (particle diameter smaller than 20 μm) on the other hand should have several advantages in terms of lower fluidization velocity required and higher particle to gas and surface-to-bed heat transfer and mass transfer rate;^37^ nonetheless, fluidization is dominated by cohesive interactions, making a smooth fluidization more difficult to achieve. For finer particles with < 20 μm diameter belonging to the group C of the Geldart classification, van der Waals interactions dominate the hydrodynamic forces in the fluidized bed.^27−29^ Agglomerates form in situ, broadening the actual size dispersion of the particles, so that they do not fluidize smoothly, and gas channeling through the bed takes place. At the higher gas velocities required to fluidize the agglomerates, finer particles can more easily be elutriated and escape the reactor.^28^ Therefore, despite its potential, CVD on a fluidized bed of fine powders is made more difficult and requires more ingenuity in reactor design to avoid particle agglomeration and entrainment.

In this contribution, we developed two methods for the deposition of inorganic semiconductor coatings of tin dichalcogenides on HC fine powders, based on plug flow- and FB-CVD. The fluidization of HC fine powders was achieved by optimizing the FB-CVD reactor design in order to minimize the particles entrainment. A custom-built bulb-shaped quartz reactor was designed to optimize the CVD from precursors in the vapor phase. The single-source precursors [SnCl_4_{^*n*^BuSe(CH_2_)_3_Se^*n*^Bu}] and [SnCl_4_{^*n*^BuS(CH_2_)_3_S^*n*^Bu}]^40,41^ were dissolved in a volatile solvent, and the solution was injected directly in the fluidized hard carbon powders bed at high temperature, with the advantage of having high flows of precursor compared to a gaseous counterpart, whose feed rate would be limited by the saturation capacity of the carrier gas.^35^ As a consequence, reaction times could be reduced, preventing the attrition of the deposited layers. Composites formed in all of the conditions explored, but the quartz reactor design and the injection conditions had to be optimized to retain the fine powders, which are subject to agglomeration and entrainment, in the reactor and maximize the deposition. Changes in the reactor design played a key role in optimizing powder retention and producing composites with a uniform composition. This study on HC powders could be extended to other C-based support materials and inorganic nanomaterials and pave the way for a more generalized method for the synthesis of powder composites.

It is worth mentioning that FB-CVD has so far been used for the synthesis of metals, alloys (FeAl, NiAl), metal oxides, such as SiO_2_, SnO_2_, ZrO_2_, TiO_2_, Al_2_O_3_,^30−34,36^ carbides,^38^ and nitrides.^39^ To the best of our knowledge, this is the first example of FB-CVD of metal chalcogenide semiconductors. The use of single source precursors was crucial for the deposition of uniform thin films dichalcogenides with controlled composition and stoichiometry, since once the complex dissociates at high temperature both Sn and either Se/S become available in a specific ratio, and their reactivity is spatially resolved.^40,41^ We propose that this method could be further extended to other semiconductors and their combination to form materials with a tunable band gap and energy density.

## Experimental Section

### Materials

Hard carbon powders Carbotron P(J) were provided by Kureha Battery Materials Japan Co., Ltd. Before use, they were dried at 70 °C for 24 h and 120 °C for 2 h to eliminate adsorbed moisture. The solvents used (THF, toluene, and hexane) were dried by distillation from sodium wire prior to use.

**[SnCl**_**4**_**{**^**n**^**BuSe(CH**_**2**_**)**_**3**_**Se**^**n**^**Bu}]** precursor was prepared according to de Groot et al.^40^ Selenium granules were ground into a fine powder under nitrogen atmosphere for 5 min and loaded in a Schlenk tube under inert atmosphere together with 25 mL of anhydrous THF and a magnetic stirrer bar. The tube was connected to a Schlenk line to be purged under a nitrogen atmosphere and placed on a stirring plate and into a liquid nitrogen bath to freeze the solution. A dry solution of ^*n*^BuLi in hexanes (15 mL, 2.43 × 10^–2^mol) was then injected dropwise through a septum. The liquid nitrogen bath was removed, and the system was allowed to stir. Further, 1–2 mL ^*n*^BuLi solution was added dropwise until a transparent pale-yellow solution was obtained, indicating that all the Se had reacted. The solution was allowed to stir at room temperature for 1 h. A total of 1.5 mL of 1,3-dichloropropane was added dropwise under stirring at room temperature and allowed to stir for 2 h. The solution was concentrated in vacuo, washed in hexane (15 mL), and filtered to eliminate the LiCl precipitate. The filtered solution was dried in vacuo to recover a pale-yellow oil which was stored in a N_2_-filled glovebox.

For the precursor synthesis, 0.9 g of SnCl_4_ was loaded in a Schlenk tube under inert atmosphere and dissolved in 15 mL of anhydrous hexane. A solution of 1.06 g of ^*n*^BuSe(CH_2_)_3_Se^*n*^Bu dissolved in 10 mL of dry hexane was injected dropwise in the SnCl_4_ solution while stirring for 30 min. The precursor was then washed with hexane, filtered, and dried in vacuo to eliminate all the solvent. The powder (yield ≈ 60%) was collected and stored in a nitrogen-filled glovebox.

**[SnCl**_**4**_**{**^**n**^**BuS(CH**_**2**_**)**_**3**_**S**^**n**^**Bu}]** was synthesized according to Gurnani et al.^41^ For the precursor synthesis, a solution of 1 mmol of ligand in 5 mL of anhydrous hexane was added slowly to a solution of SnCl_4_ 1.25 mmol in anhydrous hexane (10 mL) under constant stirring at room temperature and under a dinitrogen atmosphere. After 20 min, the white precipitate was dried in vacuo and stored in a glovebox.

### Low Pressure-CVD (LP-CVD)

Precursor powders (30 mg) were loaded in a nitrogen filled glovebox at the closed end of a quartz tube together with Si/SiO_2_ (500 nm) substrate tiles (each 20 × 8 × 1 mm), which were positioned adjacently along the tube at regular space intervals from the precursor. Two glass valves at both ends of the tube allowed it to be closed under a dinitrogen atmosphere before moving it to a Schlenk line.

The tube was then placed in the middle of a tubular furnace, the precursor being initially just out of the furnace and connected to a Schlenk line. The system was kept under a vacuum until the pressure stabilized at 0.2 mmHg. Quartz wool was placed at both ends of the furnace to secure a stable temperature inside the furnace. The furnace temperature was then set to the desired temperature. Once the temperature was stable, the tube was moved inside the furnace so that the bulb, containing the precursor, would be inside the furnace. The system was kept at high temperature for 30 min and successively taken out of the furnace to cool fast. When at room temperature, it was transferred into a glovebox.

A temperature profiling was accomplished with a separate probe to register the actual substrate temperature in different zones of the furnace.

### Plug Flow-CVD

**Figure 1:**
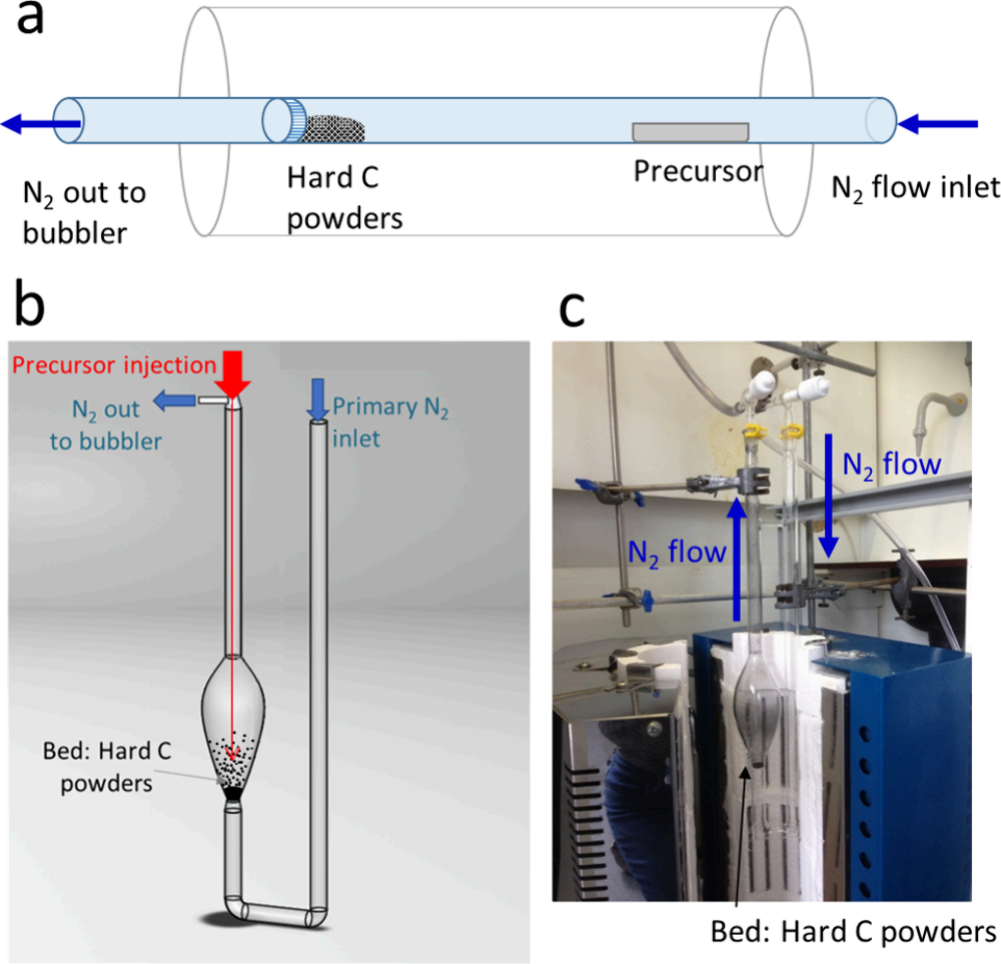
(a) Sketch of the plug flow-CVD setup; (b) sketch of FB-CVD reactor; (c) picture of the FB-CVD setup.

A sketch of the setup is shown in Figure 1a. HC powders (80 mg) were loaded in an open quartz tube adjacent to a disk of sintered quartz. The solid precursor, in the form of a powder (30 mg), was loaded in a boat in a nitrogen filled glovebox and placed at the other end of the quartz tube. Two valves at opposite ends of the tube were closed to secure the tube under a nitrogen atmosphere. The tube was then adjusted in the middle of a tubular furnace so that HC powders were in the heating zone and the precursor would be out of the furnace. The tube was connected by one end to a Schlenk line and by the other to a bubbler. Nitrogen was allowed to flow for a few minutes, and the furnace was heated to the reaction temperature, i.e., 400, 450, 500, and 600 °C, in the respective experiments. Once the temperature was stabilized, the tube was moved in such a way that both the precursor and HC substrate were in the heating zone of the furnace. After 30 min at high temperature, the tube was moved out of the furnace and allowed to cool down. Once at room temperature, the tube was brought to a reduced pressure and transferred in a glovebox, where the powders were collected and stored. The HC-precursor distance was also adjusted to regulate the precursor evaporation temperature and the deposition temperature.

### FB-CVD

A sketch and a picture of the setup, prior to modifications, are shown in Figure 1b,c. In a typical experiment, 0.2 g of precursor was loaded in a Schlenk tube under a flow of nitrogen and dissolved in 12 mL of anhydrous toluene by slightly heating to 50 °C under stirring for 10 min. A total of 0.8–1 g of hard carbon powders (average diameter 10 μm) was loaded on the FB-CVD reactor distributor plate, and the nitrogen flows were adjusted to 0.3 L min^−1^. Under these conditions, the powders were slightly moving on the plate, but a full fluidization was not achieved. The temperature was increased by 20 °C/min up to 450 °C, with the bulb of the reactor being in the middle of the heating zone of the tubular furnace. Once the temperature was stabilized, the nitrogen flows were increased to 0.5−0.8 min^−1^ to achieve a full fluidization of the powders. The air-free precursor solution was withdrawn with a syringe and injected dropwise (1.3 mL min^−1^) with the aid of a syringe pump through a septum directly in the CVD zone. The system was kept at high temperature for an additional 30 min after all the precursor was injected and then allowed to cool to room temperature. Alternatively, for a multiinjection process, a second injection of precursor was performed in the same conditions, then kept steady for 30 min before cooling down.

When at room temperature the reactor was closed under nitrogen and transported to a nitrogen-filled glovebox, where the powders were collected.

### Characterization Methods

^1^H and ^13^C NMR spectra were measured from solutions in anhydrous CDCl_3_ on a Bruker AV400 spectrometer. The analytical data are the following.

[SnCl_4_{^n^BuSe(CH_2_)_3_Se^n^Bu}] (Figure S1). ^1^H NMR (CDCl_3_, 298 K) δ/ppm: 0.92 (t, [6H], CH_3_), 1.48 (m, [4H], CH_2_CH_3_), 1.75 (m, [4H], CH_2_), 2.45 (m, [2H], –SeCH_2_CH_2_CH_2_Se−), 3.22 (m, [4H], CH_2_Se), 3.32 (m, [4H], SeCH_2_). ^13^C{^1^H} NMR (CDCl_3_) δ/ppm: 13.41 (CH_3_), 22.83 (CH_2_), 25.02 (CH_2_), 30.05 (CH_2_), 32.9 (CH_2_), 35.58 (CH_2_).

[SnCl_4_{^n^BuS(CH_2_)_3_S^n^Bu}] (Figure S2). ^1^H NMR (CDCl_3_, 298 K) δ/ppm: 0.95 (t, [6H], CH_3_), 1.43 (m, [4H], CH_2_CH_3_), 1.72 (m, [4H], CH_2_), 2.31 (m, [2H], –SCH_2_CH_2_CH_2_S−), 3.07 (m, [4H], SCH_2_), 3.26 (m, [4H], SCH_2_). ^13^C{^1^H} NMR (CDCl_3_) δ/ppm: 13.70 (CH_3_), 21.77 (CH_2_), 29.40 (CH_2_), 34.19 (CH_2_), 36.74 (CH_2_).

X-ray diffraction (XRD) patterns were collected by using a Rigaku SmartLab system. Samples synthesized by LP-CVD on Si/SiO_2_ substrates were studied in grazing incidence geometry with X-ray Cu Kα (λ= 1.5418 Å) and a Hypix 2D detector. HC-based samples synthesized by plug-flow and FB-CVD were analyzed by capillary transmission. XRD of the TGA residue powders was measured with a Bruker D2 Phaser instrument using Cu Kα radiation. Scanning electron microscopy (SEM) was conducted using a Philips XL-30 ESEM (20 kV accelerating voltage) with a ThermoFisher Ultradry energy dispersive spectroscopy (EDS) detector and Noran System 7 data processing. A ZEISS sigma FE-SEM was used for the imaging and elemental mapping of multimaterial composites (HC-SnS_*x*_–SnSe_2_). Thermogravimetric analysis (TGA) used a Netzch TG 209 F1 Libra instrument with a heating rate of 5 °C min^–1^ up to 1100 °C under 20 mL min^–1^ of oxygen and 30 mL min^–1^ of argon and a protective Ar flow of 20 mL min^–1^. The BET surface area and porosity were analyzed by nitrogen physisorption with a BET-TriStar II 3020.

### CFD Study

The geometry of the reactor was designed with SolidWorks. The HC powders were approximated to spherical C particles with a density of 1.5 g cm^–3^^42^ and average particle size of 10 μm, as obtained from a size statistics measured by SEM images with an Image J software.

### Governing Equations

The kinetic theory of granular flow (KTGF) model within Ansys Fluent 2021 R2 was used to model the interactions between gas and solid particles within this fluidized bed. This model considers two different phases in one controlled volume by applying a volume fraction variable and treats them as an interpenetrating continuum. The carbon solid phase was assumed to consist of spherical particles of the same diameter of 10 μm. Each phase was solved individually in accordance with the mass and momentum conservation equations, as presented in Table 1. The gas–solid interphase exchange coefficient, *K*_gs_, was modeled using the Syamlal–O’Brien drag model.^43^ No reactions were considered in the CFD model, and the inlet temperatures and reactor temperatures are assumed to have reached an isothermal temperature of 430 °C. The gaseous phase properties were taken accordingly at that temperature, and the energy equation is neglected.

**Table 1:** Breakdown of the Governing Equations

**conservation of Mass**		
gas		(1)
solid		(2)

**conservation of momentum**		
gas		(3)
solid		(4)
phase stress–strain tensor for phase, _*i*_		(5)

**momentum interphase exchange coefficient**		
Syamlal–O’Brien drag model		(6)
		(7)
		(8)
	; for ε_N2_ > 0.85	(9)
	; for ε_N2_ ≤ 0.85	(10)

**kinetic theory of granular flow**		
kinetic fluctuation energy		(11)
		(12)
		(13)
	φ_gs_ = – 3*K*_gs_θ_c_	(14)
phase definitions	ε_N2_ + ε_c_ = 1	(15)

The kinetic fluctuations between particles were considered using the kinetic theory of granular flow given in Table 1. A full review of the equation derivation is provided by Gidaspow.^44^ The solid shear viscosity, μ_c_, is composed of collisional, μ_c, col_, kinetic, μ_c, kin_, and frictional, μ_c, fr_, interactions. For highly dense flows, such as those experienced by Geldart C particles, the frictional viscosity is applied as the volume fraction for the particles approaching the maximum packing limit. The frictional model of the Schaeffer expression^45^ was used to model the frictional viscosity in the present case. The bulk viscosity, λ_c_, accounts for the resistance of particle to expansion and depression and was calculated using an expression from Lun et al.^46^ The solid pressure, *p*_c_, considers both kinetic and collisional contributions and evolves from an equation of state that is analogous to the van der Waals equation of state for gases.^47^ The radial distribution function, *g*_0_, modifies the probability of particle collisions as the phase approaches the maximum packing limit. Table 2 provides an overview of the constitutive equations used in the present case.

**Table 2:** Breakdown of the Constitutive Equations

solid shear viscosity	μ_c_ = μ_c, col_ + μ_c, kin_ + μ_c, fr_	(16)
collisional viscosity		(17)
kinetic viscosity		(18)
frictional viscosity		(19)
solid bulk viscosity		(20)
solid pressure		(21)
radial distribution function		(22)

#### Boundary and Initial Conditions

The particle bed is initially set to a height of 0.01 m above the quartz disc distributor. The particles were assigned a diameter, *d*_c_ = 10 μm, and density, ρ_c_ = 1520 kg m^–3^. The inlet provided a continual supply of nitrogen with cases considering velocities ranging from 0.1 m s^–1^ <  < 0.2 m s^–1^ to reflect a flow rate spanning 0.5–1.0 L min^–1^. A pressure outlet was used and set to atmospheric pressure. The particle–wall collisions significantly impact the shear stress on the walls of the bulb reactor. No-slip boundary conditions were assigned for the gas phase tangential and normal velocities, whereas a tangential slip condition is imposed for the particulate phase which was developed by Johnson and Jackson.^48^ The near-wall granular temperature considers the granular temperature flux normal to the wall and to the energy dissipation due to particle-wall collisions. A breakdown of the gaseous and particulate physical properties is provided in Table 3, along with key closure parameters to support the collisional equations provided in Tables 1 and 2.

**Table 3:** Table of Parameters

	**parameter**	**value**
**Gas: Nitrogen**		
	velocity	0.1 – 0.2 m s^–1^ (∼0.5 −1.0 L min^–1^)
ρ_*N*2_ (@ 450 °C)	density	0.469 kg m^–3^
μ_N2_ (@ 450 °C)	shear viscosity	3.283 kg m^–1^ s^–1^
**Particle: Carbon**		
ρ_c_	particle density	1520 kg m^–3^
*d*_c_	particle diameter	10 μm
*e*	particle coefficient of restitution	0.95
*e*_w_	wall coefficient of restitution	0.95

#### Numerical Simulations

The finite volume method was used to solve the governing equations. The coupling and correction of the velocity and pressure was carried out using the Phase Coupled SIMPLE (PC-SIMPLE) algorithm.^49^ The discretization of the convective terms was performed by using the second-order upwind scheme. An unsteady simulation was performed with a time step of 1 × 10^–4^ s to ensure fast convergence with 30 iterations per time step. The convergence criterion between two iterations was set to 1 × 10^–3^.

## Results and Discussion

### Optimization of CVD Conditions

To test the optimal conditions for the deposition of tin dichalcogenides, we carried out LP-CVD from tin seleno- and thio-ether complexes on Si/SiO_2_ substrates at different temperatures. In each experiment, several substrates could be placed in different positions of the furnace, i.e. experiencing different temperatures, so that it was possible to establish the optimal deposition temperature. Deposition from [SnCl_4_{^*n*^BuSe(CH_2_)_3_Se^*n*^Bu}] at temperatures of 400 °C and higher produced SnSe_2_ coatings consisting of randomly oriented hexagonal crystallites whose structure is consistent with bulk SnSe_2_ (Figure S3, top panels). SnS_2_ coatings were deposited from [SnCl_4_{^*n*^BuS(CH_2_)_3_S^*n*^Bu}] at temperatures in the range of 310–350 °C. In the latter experiments, the precursor was heated to temperatures as high as 650 °C, with the deposition taking place in the colder zone of the furnace, indicating that faster precursor evaporation produced uniform crystalline SnS_2_ coatings (Figure S3, bottom panels). The coating composition was confirmed by EDS spectra acquired in correlation with SEM, measured at different locations on the thin films surface, showing ratios of Sn to S close to 1:2 (Figure S4).

### Tin Dichalcogenide-HC Composites by Plug Flow-CVD

A different configuration was then used to deposit SnSe_2_ and SnS_2_ on hard carbon powders by plug flow-CVD under a dinitrogen flow (Figure 1a). These experiments aimed at determining the best conditions for CVD on HC powder substrates under dinitrogen flow ambient pressure. The samples obtained after CVD from [SnCl_4_{^*n*^BuSe(CH_2_)_3_Se^*n*^Bu}] were analyzed by XRD and SEM/EDS. All samples showed a high yield of deposited tin chalcogenide, but its distribution was uneven. While some particles of the powders were completely coated with tin chalcogenide platelets, some others were completely free of any sign of SnSe_2_. Sample homogeneity could be improved to a certain extent by using small amounts of HC (80 mg) and spreading the powder on the tube surface to allow most of the HC grains’ surface to be exposed to the precursor vapors. Overall, samples obtained in the deposition temperature in the 380–450 °C range gave the best results in terms of amount and homogeneity of SnSe_2_ deposited on HC (Figure S5). Slower precursor evaporation, achieved by a larger distance of the precursor source from the furnace hot zone, increased the amount of deposited material.

Plug-flow CVD from [SnCl_4_{^*n*^BuS(CH_2_)_3_S^*n*^Bu}] on HC exhibited greater sensitivity to temperature than LP-CVD. Samples obtained at deposition temperatures in the range of 300–450 °C showed the presence of SnS_*x*_ species deposited on the surface of HC (SEM-EDS), but XRD analysis revealed a poor crystallinity. By increasing the furnace temperature to 600 °C and increasing the precursor–substrate distance, as in the previous experiments at low pressure, SnS was predominant (Figure S6).

TGA of the samples under O_2_/Ar showed a considerable mass loss at temperatures higher than 550 °C due to the C combustion, and the XRD analysis of the residue showed the complete conversion of SnSe_2_ into SnO_2_ (Figure S7a,f). The SnSe_2_ content of the samples was calculated from this data to be around 15–30 wt%.

### Tin Dichalcogenide-HC Composites by FB-CVD

In order to scale up the process to the gram scale and further improve the uniformity of the coatings, we developed a FB-CVD strategy. We designed a quartz reactor as in Figure 1b,c, consisting of a bulb-shaped reaction zone and a disk of sintered glass as the reactor bed, functioning as a distributor, where the HC powders were loaded. The main nitrogen flow passed through a quartz tube from the bottom of the reactor through the distributor, applying pressure to the HC powders. The reactor was placed in a vertical tubular furnace so that the whole bulb would be within the hot zone of the furnace. Once stabilized at the reaction temperature and under nitrogen flow, the precursor, as a solution in toluene, was dropwise injected with a syringe via a long needle, at 2 cm from the top of the distributor. A syringe pump was used to control the injection rate.

**Figure 2:**
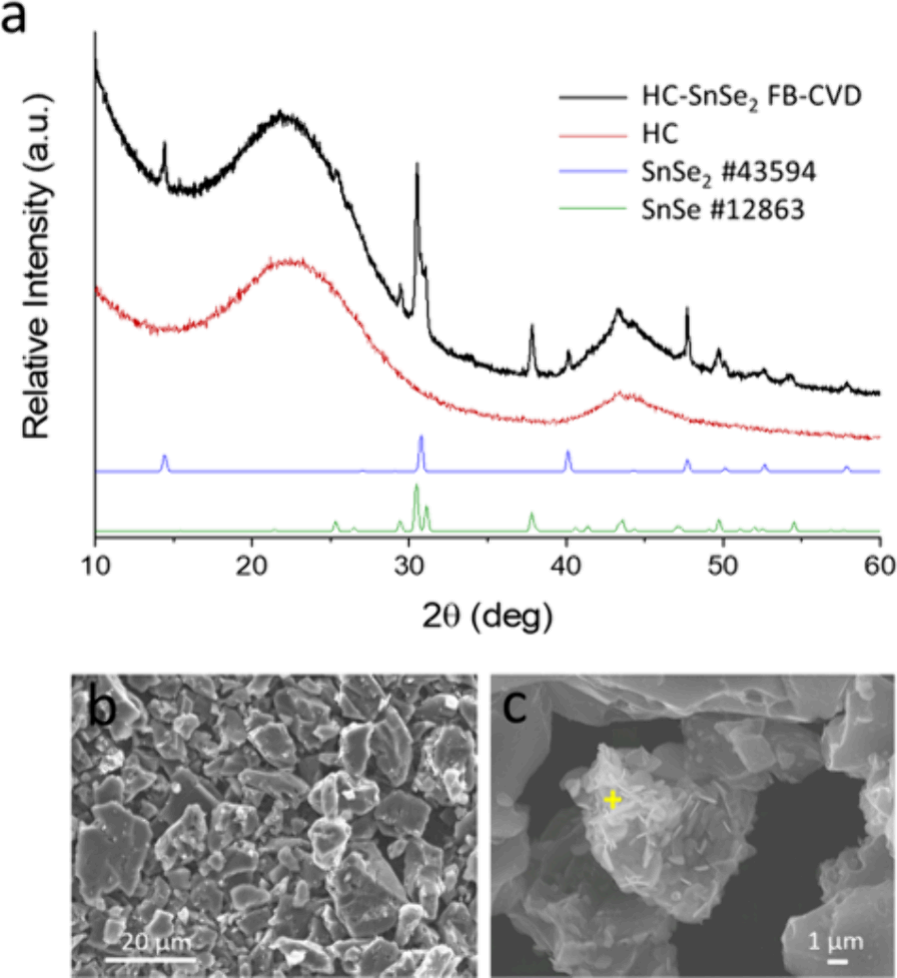
(a) Powder XRD by capillary transmission of an HC-SnSe_2_ sample obtained by FB-CVD at 430 °C and SEM micrographs obtained at lower (b) and higher (c) magnification.

In the first instance, we focused on the deposition of SnSe_2_ from [SnCl_4_{^*n*^BuSe(CH_2_)_3_Se^*n*^Bu}] on HC powders in a FB-CVD set up at different temperatures and flows. Figure 2 shows samples obtained after 40 min deposition at 430 °C, consisting of SnSe_2_ flakes on the surface of HC, as observed from SEM analysis. EDX acquired at different locations showed consistently a Sn:Se ratio close to 1:2. EDX acquired at the spot indicated in Figure 2c (yellow cross) showed the following composition: Se L 5.01 at. %, Sn L 2.81 at. %, C K 92.18 at. %. The composition was confirmed by XRD (Figure 2a), showing sharp 2H-SnSe_2_ peaks. A minor component of SnSe was observed by the appearance of a peak at 37.9°. In the preliminary conditions explored, we consistently observed a significant loss of material by entrainment. Compared to plug flow CVD, the powders are very mobile in the fluidized bed and are not completely confined to the reaction zone. A finer component of the powders can move to the higher part of the reactor, where lower temperatures are experienced so that some deposition can take place in this colder zone of the furnace. The percentage of SnSe_2_ was determined by TGA under an atmosphere of Ar and O_2_ showing a mass loss around 600 °C. XRD of the TGA residue confirmed a complete conversion to SnO_2_ under these conditions and the sample composition was calculated to be in the order of 7 wt% SnSe_2_ (Figure S7b).

These results demonstrated that the high temperature injection of a precursor solution in a fluidized powder bed is a viable route for the synthesis of metal-chalcogenide-coated carbon composite materials. Nevertheless, the fluidization was not stable but bubbling and turbulent, leading to substantial entrainment. This behavior was expected for the HC, which consisted of fine powders with an average 10 μm grain size and low density, hence belonging to the Geldart group C classification.^27,29^ For such particles, cohesive van der Waals forces prevail, causing agglomerates to form in situ and substantially broaden the actual particle size distribution. Bigger agglomerates require higher gas velocities to be fluidized, but at such higher gas velocities, smaller particles can escape the reactor. The coexistence of particles of different sizes thus makes smooth fluidization difficult.

### CFD Study

**Figure 3:**
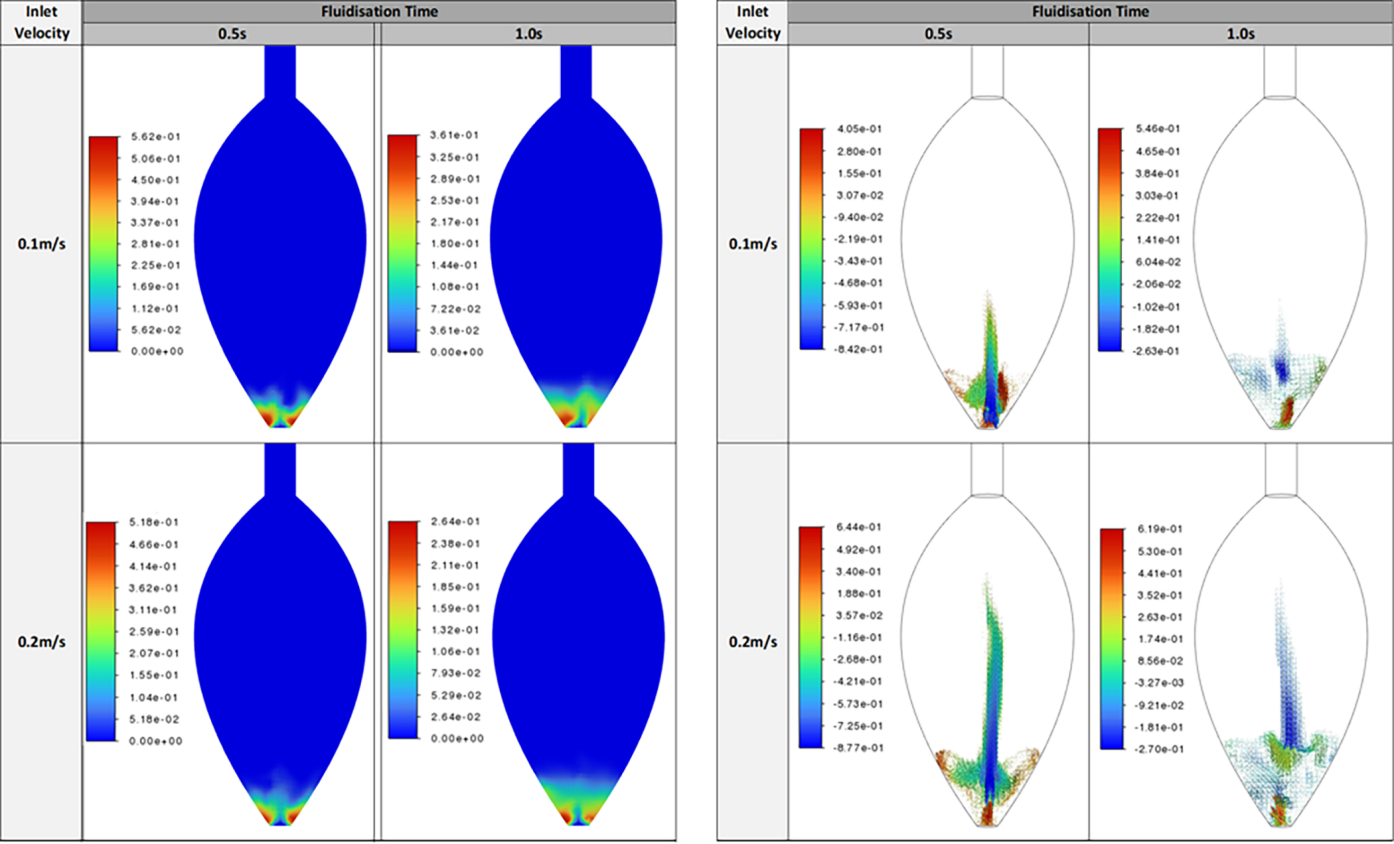
CFD comparison study of the volume fraction (left panels) and of the *y*-velocity (right panel) of carbon for different nitrogen inlet velocities along the central plane of the reactor after 0.5 and 1.0 s.

A CFD evaluation was conducted to evaluate the gaseous and solid flow dynamics within the proposed reactor. For the simulation, HC powders were represented as spherical particles of 10 μm diameter with a density of 1.52 g cm^–3^. Simulations were run at gas velocities of 0.1, 0.15, and 0.2 m s^–1^ to identify the range in which the powders are fluidized. Figure 3 (left panels) shows a comparative study of the carbon volume fraction at different gas velocities of inlet N_2_ in the first seconds, i.e., after 0.5 and 1.0 s for nitrogen inlet velocities 0.1 and 0.2 m s^–1^ along the central *XY*-plane of the bulb reactor. For both inlet velocities, the particle bed resides in the lower region of the bed with denser packing observed toward the annular region of the reactor and a dilute core. This is expected given that these Geldart C particles have high frictional stresses due to high interparticle forces.^29^ The cohesive nature of such small particles demonstrates the agglomeration of particles and the creation of a gas-channel through the center of the reactor. For both inlet velocities, the maximum volume fraction is higher at 0.5 s than at 1.0 s, indicating that the bed had yet to reach fluidized state. This is reinforced with a slightly higher bed height observed at 1.0 s compared with 0.5 s. Increasing the inlet velocity to 0.2 m s^–1^ further aerates the particles, promoting a more dilute flow of the particles which can observed at both 0.5 and 1.0 s of fluidization time.

The *y*-velocity directional component of the carbon across the central *XY*-plane is presented in Figure 3 (right panels) and shows more clearly the extent of carbon distribution within the bulb reactor for the different nitrogen inlet velocities, 1.0 and 0.2 m s^–1^. Given that this is the velocity specifically captured in the *y*-direction, it is clear that there are particles upward, i.e., *v*_p_ > 0 m s^–1^, and particles traveling back toward the inlet, i.e., *v*_p_ < 0 m s^–1^. Cross-referencing against Figure 3 the *y*-velocity distributions suggests that the particles travel further into the freeboard of the reactor than the volume fraction contours suggest.

Initial observations show a faster upward velocity near the gas inlet. A significant particle presence is observed above the central part of the bed, which lies above the central gas channel. Closer inspection of Figure 3 suggests that these particles are traveling downward and, moreover, reducing in occurrence as the fluidization time increases. This is very likely attributed to the expulsion of particles that occurs once the gas-channel breaks through the bed. Figure S8 shows an expansion of the negative and positive *y*-velocities in the run up to the 0.5 s point, specifically at 0.2 0.3, and 0.4 s for a nitrogen inlet velocity of 0.1 m s^–1^. At 0.2 s, the particles are traveling upward with a greater magnitude than those descending. There is a clear circulation to the flow in the annular of the reactor with a central upward flow of particles driven by the gas traveling through the gas channel. By 0.3 s, there is a great downward velocity as many of the particles return to the bed. The maximum upward velocity is also lower, which is expected as the flow responds to the freeboard and the divergent geometry of the reactor. These trends continue for the downward and upward flow at 0.4 s and the magnitudes at 0.5 s in Figure 3 for 0.1 m s^–1^, although the particles exhibiting a positive *y*-velocities at 0.4 s do not travel as high into the freeboard, whereas those that are in the freeboard are descending.

A further observation is that at 0.3 and 0.4 s in Figure S8, and at 0.5 s in Figure 3, there is an increasing presence of particles with a positive *y*-velocity nearer the annular near the walls. This will likely be due to walls offering a more favorable means for the gas to traverse around the denser particle regions, which will have the higher interparticle forces. It was previously noted in Figure 3 at 1.0 s that for both the 0.1 and 0.2 m s^–1^ inlet velocities the particle bed appeared more dilute and aerated, and this is further observed with the *y*-velocity distributions in Figure 3 with a slightly higher bed expansion and slightly more circulatory flows.

Positive and negative velocities (Figures S9 and S10) furthermore show that there is entrainment of particles early in the process, as a response to the gas breaking through the bed and the gas channeling into the freeboard with a great inlet velocity. After 0.4 s, it is clear that slower particles have returned to the base of the reactor and fluidization continues. The close up in Figure S10 better shows the extent of mixing within the particle bed. Circulatory flow is evident, with the flow driven from faster flow at the walls and particles rolling back into the core of the reactor. Downward particles come from the freeboard, where particles have dropped back down to the bed, while the central core provides a faster stream of particle flow in response to the fast inlet velocity through the center of the bed.

### Optimal FB-CVD Reactor Design for the Synthesis of Tin Dichalcogenide-HC Composites

**Figure 4:**
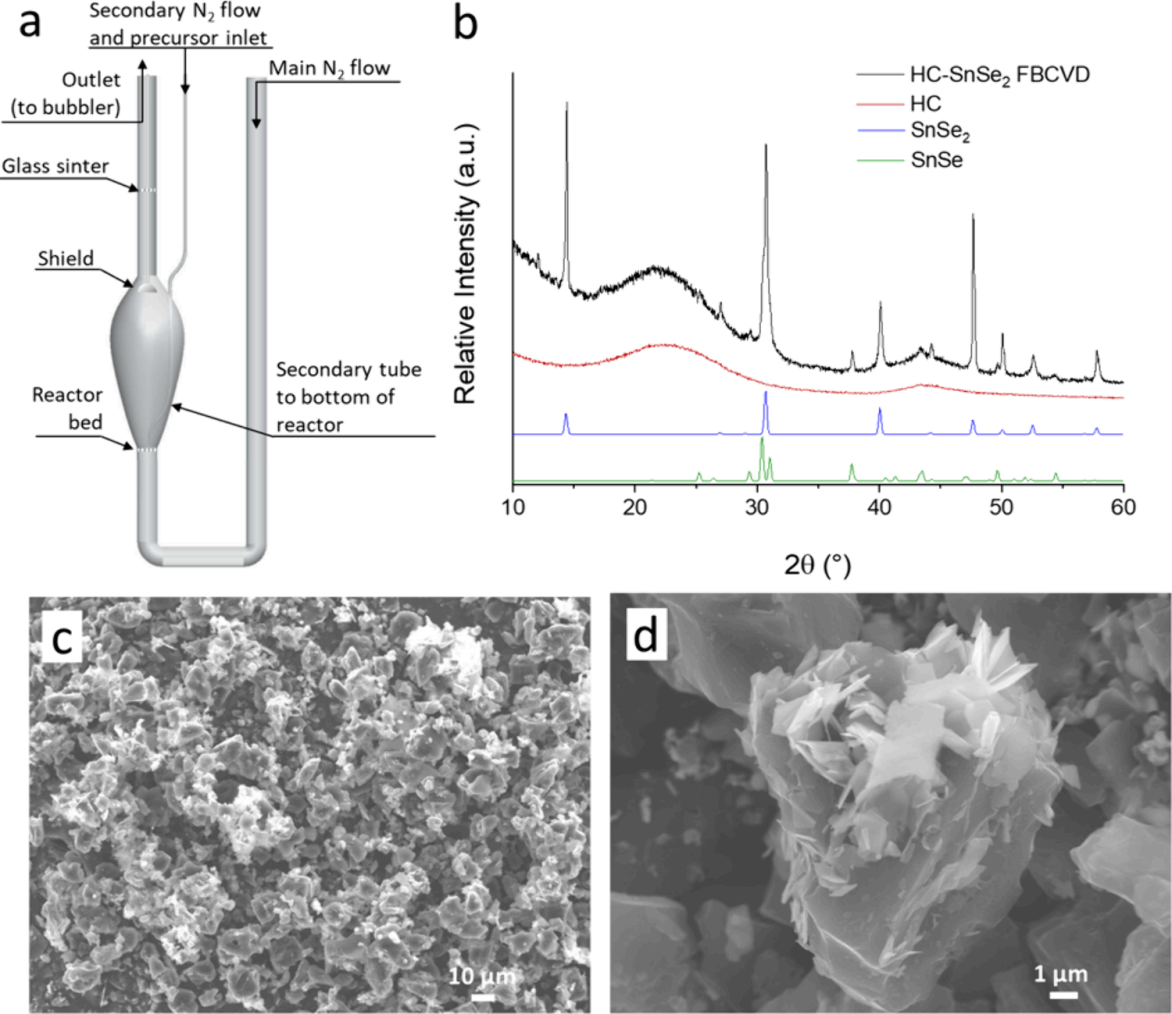
(a) Sketch of the modified FB-CVD reactor; (b) XRD; lower (c) and higher (d) magnification SEM micrographs and of a HC-SnSe_2_ sample obtained by FB-CVD at 430 °C.

This study confirmed that at the gas velocities of 0.15 m s^–1^ used in our method, there is a significant entrainment of carbon particles. It also suggests that gas velocities as low as 0.1 m s^–1^ may allow a more significant confinement of the particles in the reactor’s bed and a sufficient bed expansion to allow FB-CVD. For these reasons, we focused on lower gas velocities of the inlet N_2_ but aimed at improving the fluidization by modifying the reactor geometry and by aiding powder mixing. As shown in Figure 4a, a thin quartz tube with 1 mm tip diameter was introduced, allowing addition of a secondary inlet of nitrogen flowing directly on the reactor bed, to improve powder mixing and to avoid the formation of agglomerates. The precursor was also injected through this thin tube and transported to the reactor bed. A (removable) shield and a sintered quartz filter were added to the top of the main outlet to better retain the powders within the reaction zone. The main and secondary nitrogen flows were regulated to 0.5 L/min. These measures allowed retention of 30 to 50% of the HC powders.

Samples obtained by FB-CVD with the modified reactor from the [SnCl_4_{^*n*^BuSe(CH_2_)_3_Se^*n*^Bu}] precursor on 1 g of HC, at 430 °C, are shown in Figure 4b–d. XRD shows the clear presence of SnSe_2_ and a smaller contribution from SnSe (Figure 4b). SEM at lower magnification evidence the presence of high electron contrast areas uniformly distributed on the powder surface (Figure 4c). At higher SEM magnification, it is possible to distinguish SnSe_2_ flakes, with high electron contrast and a typical planar structure, emerging from the surface of HC, showing the formation of a direct HC-SnSe_2_ interface. A 13 wt% loading of SnSe_2_ was measured by TGA in Ar/O_2_ with the residue consisting of SnO_2_ (Figure S7c).

N_2_ adsorption–desorption isotherms of the HC-SnSe_2_ samples obtained by FB-CVD were acquired and compared to those of pure HC. HC-SnSe_2_ showed a higher specific surface area of 12 m^2^ g^–1^ compared to 2.3 m^2^ g^–1^ of untreated HC (Figure S11). The increased surface area of the composites could be attributed to their higher surface roughness, which is also evident by observing the composites’ morphology at SEM. All samples showed a broad pore size distribution (Figure S12), which is dominated by the particles packing rather than an intrinsic porosity.

### Synthesis of SnS_*x*_–SnSe_2_ layers on HC

**Figure 5:**
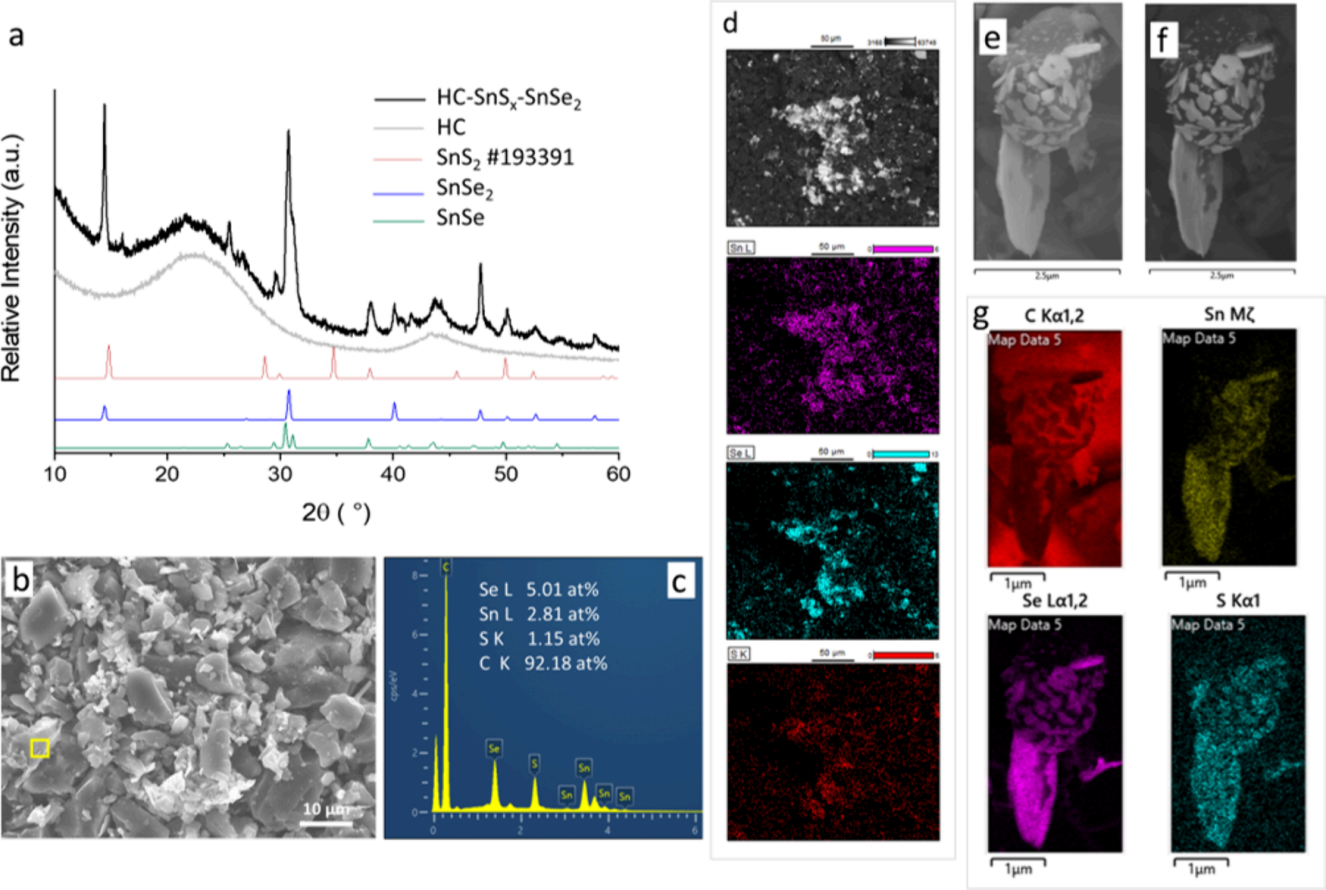
(a) Capillary transmission XRD, (b) SEM, (c) EDS, and (d) elemental mapping of HC-SnS_*x*_–SnSe_2_ synthesized by FB-CVD by multiple injections. (e, f) High resolution SE micrographs of a single particle, also acquired with a backscattering detector (f), and (g) corresponding elemental mapping.

In order to make layers of the different materials, we performed two separate injections of the two precursors. A first dropwise injection of the [SnCl_4_{^*n*^BuS(CH_2_)_3_S^*n*^Bu}] precursor was performed at 350 °C, and after 30 min, the temperature was raised to 430 °C, at which the [SnCl_4_{^*n*^BuSe(CH_2_)_3_Se^*n*^Bu}] precursor solution was injected dropwise. XRD showed the prevalence of SnSe_2_ and a minor contribution of SnSe, while the presence of either SnS_2_ or SnS was not evident. Nevertheless, the presence of Sn, Se, and S species was confirmed by SEM/EDS, with a ratio of metal to chalcogen close to 1:2, while the absence of Cl-containing species ruled out the presence of unreacted precursors (Figure 5b,c). TGA showed a residual mass comparable to or higher than the one observed for the HC-SnSe_2_ samples (Figure S7d).

N_2_ adsorption–desorption isotherms of the HC-SnS_x_–SnSe_2_ samples showed a specific surface area of 5.6 m^2^ g^–1^ t, higher than untreated HC but lower than single deposit HC-SnSe_2_ composites (Figure S11). A possible explanation is that the second material deposits by filling the gaps in the original material and could partially account for the lower crystallinity. The SEM study also shows samples with slightly different morphology and surface roughness (Figure 5b,e,f).

Elemental mapping at low magnification showed a clear presence of Sn, Se, and S in the same areas with similar intensity, indicating that the species are deposited uniformly throughout the sample (Figure 5d). The absence of clear SnS_2_ or SnS peaks in the XRD analysis could possibly be due to a low crystallinity. It should be highlighted that SnS_2_ coatings previously deposited in different crystalline phases, e.g., 2H-bulk, 4H-bulk,^41^ and cubic *Fd*3̅*m* (Figure S3), were expected to show a predominant peak at 14° due to the 001 reflection, which overlaps with the 001 reflection of SnSe_2_ and could therefore be masked by a larger amount of that phase. An insight at higher magnification is shown in Figure 5e–g, displaying thin platelets protruding out of a single carbon particle, which represents a tantalizingly promising morphology for application as a battery anode active material. In light of the potential applications in electrochemistry, the high surface area reduces the proportion of electrochemically inactive material found in the center of large particles and affords a high rate capability of charge/discharge, simultaneously providing the requisite space for the metal dichalcogenide crystal lattice to expand/contract upon charge/discharge cycling of the battery, minimizing electrode cracking, and extending useful battery lifetime. This material is advantageously anchored to a high conductivity carbon core, which could facilitate electronic transport out to the current collector foil and into the electronic circuit beyond. This is more evident in the backscattering EM image in Figure 5f, where the high contrast platelets can be attributed to high electron scattering deposited species. Interestingly, the corresponding elemental mapping shows that SnS_*x*_ and SnSe_2_ were deposited in the same areas, with the possible formation of a layered structure (Figure 5g). This finding is of particular importance for novel next generation battery materials, indicating that this method can be used to fabricate multilayered composites. By choosing appropriate combinations of materials, it would be possible to engineer “metamaterials” and tune the heterolattice coatings properties, such as band-edge staggering at the interface of the inorganic layers.^24^ In particular, combination of SnS_2_ and SnSe_2_ layers on HC is a promising anode material Na-ion batteries, with the with SnS_2_-SnSe_2_ layered systems allowing for high energy density on conductive HC.^24^

## Conclusions and Outlook

Our findings show that CVD is a viable route for the synthesis of powder composites based on carbon, with controlled composition and a layered structure of tin dichalcogenides, which are extremely promising candidates for applications in batteries and electrocatalysis owing to control of morphology and the opportunity for bandgap engineering. Plug flow-CVD offered a solution to deposit metal dichalcogenides on HC powders with high deposition yield on small amounts of powders, and the process was scaled up to few hundred milligrams of material by FB-CVD. An optimized reactor configuration allowed us to minimize the natural entrainment of fine low-density powders (belonging to the Geldart C classification), with the turbulent fluidization allowing for good mixing and material uniformity. This method holds promise for generalization of the uniform CVD coating of fine powders of more different materials from either solid and liquid precursors. We observed that the retention of particles lies not only with hydrodynamics operating conditions such as velocities but also with the reactor design, with the possibility to further improve the powder retention and aim at a high nanocomposite yield. Finally, by alternating the injection of different precursors, we achieved the deposition of two different materials on the HC powders, paving the way to the fabrication on metamaterials with controlled properties such as band edge staggering and tuned energy density for applications as energy materials with important implications for the design and synthesis of novel next generation battery active materials and metamaterials.

## Supporting Information Available

^1^H and ^13^C NMR spectra of [SnCl_4_{^*n*^BuSe(CH_2_)_3_Se^*n*^Bu}] and [SnCl_4_{^*n*^BuS(CH_2_)_3_S^*n*^Bu}] precursors. XRD and SEM of SnSe_2_ and SnS_2_ thin films on Si/SiO_2_ substrates obtained by LP-CVD from single-source precursors; SEM and EDX spectra of tin dichalcogenide thin films on Si/SiO_2_ tiles obtained by LP-CVD from single-source precursors; powder XRD and SEM of HC-SnSe_2_ composites obtained by plug flow-CVD at different temperatures; TGA of composites synthesized by plug-flow CVD and FB-CVD in different conditions and powder XRD of the residue; CFD close up of the specific directional *y*-velocities along the *XY*-plane for the 0.1 m/s inlet velocity at different times; positive and negative *y*-velocities within the bulb along the *XY*-plane for the 0.4 m/s inlet velocity at varying fluidization times and comparison with the positive only *y*-velocities; close up of the positive and negative *y*-velocities along the *XY*-plane for the 0.4 m/s inlet velocity at 0.2, 0.3, 0.4, and 0.5 s BET analysis (PDF)
