# Supplementary material for: Fluidized Bed Chemical Vapor Deposition on Hard Carbon Powders to Produce Composite Energy Materials
Source: ACS Omega. 2024 Mar 7;9(11):13447–57. doi: 10.1021/acsomega.4c00297 (PMC10955755; doi:10.1021/acsomega.4c00297)
Supplement: Supplementary file 1 — ao4c00297_si_001.pdf [file ao4c00297_si_001.pdf]

SUPPORTING INFORMATION

Fluidized Bed Chemical Vapor Deposition on Hard Carbon Powders to Produce Composite Energy Materials

Marianna Casavola*, Lindsay-Marie Armstrong, Zening Zhu, Daniela Ledwoch, Matthew McConnell, Paul Frampton, Peter Curran, Gillian Reid, and Andrew L. Hector*

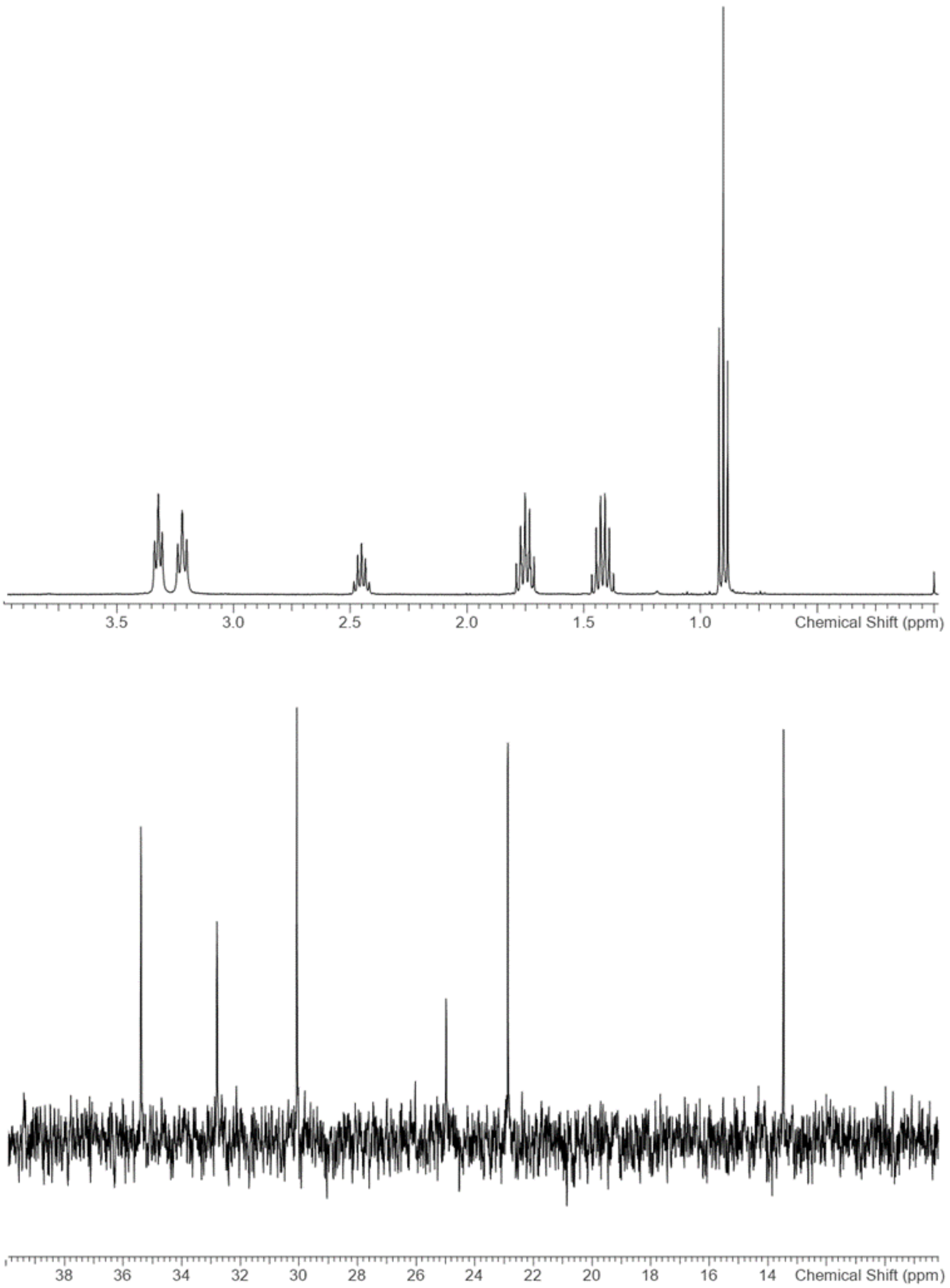

Figure S1. Room temperature ^{1}H and $^{13}C\{^{1}H\}$ NMR spectra of $[SnCl_4\{^{n}BuSe(CH_2)_3Se^{n}Bu\}]$ in $CDCl_3$.

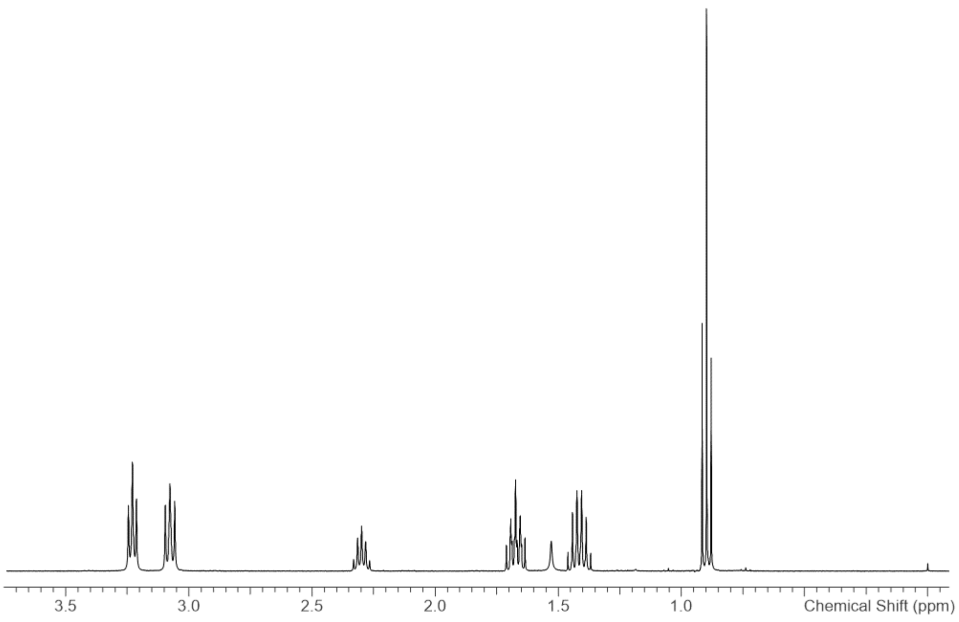

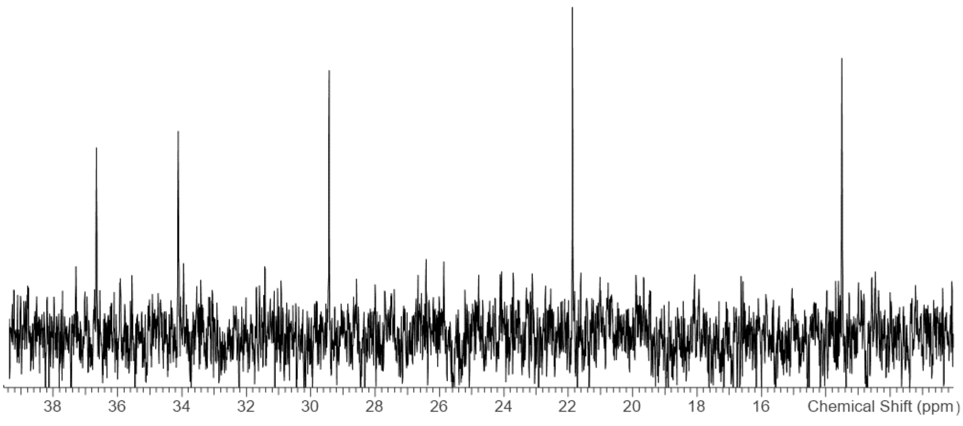

Figure S2. Room temperature ^{1}H and $^{13}C\{^{1}H\}$ NMR spectra of $[SnCl_4\{^{n}BuS(CH_2)_3S^{n}Bu\}]$ in $CDCl_3$.

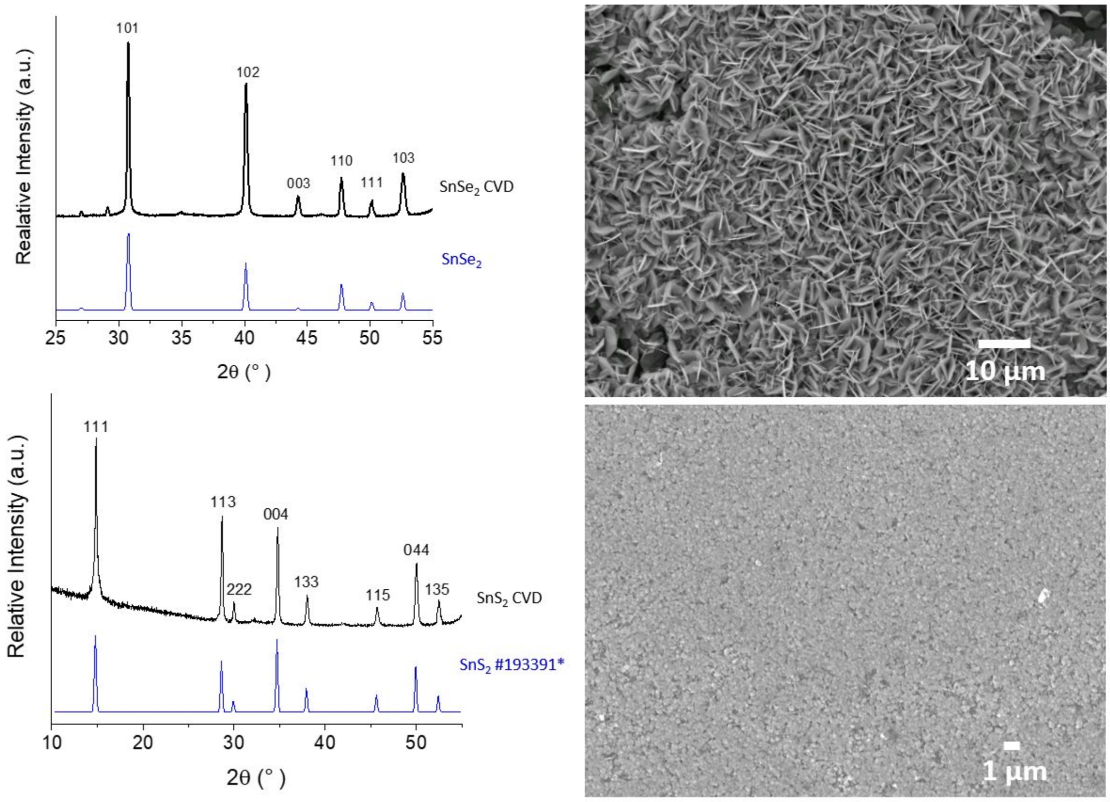

Figure S3. Top panels: XRD grazing incidence 3° (left) and SEM (right) of $SnSe_2$ thin films on Si/SiO_2 (500 nm) substrates obtained by CVD from [$SnCl_4${$^nBuSe(CH_2)_3Se^nBu$}] at 400 °C. Bottom panels: XRD grazing incidence 3° (left) and SEM (right) of SnS_2 thin films on Si/SiO_2 (500 nm) substrates obtained by CVD from [$SnCl_4${ $^nBuS(CH_2)_3SnBu$}] at 320 °C.

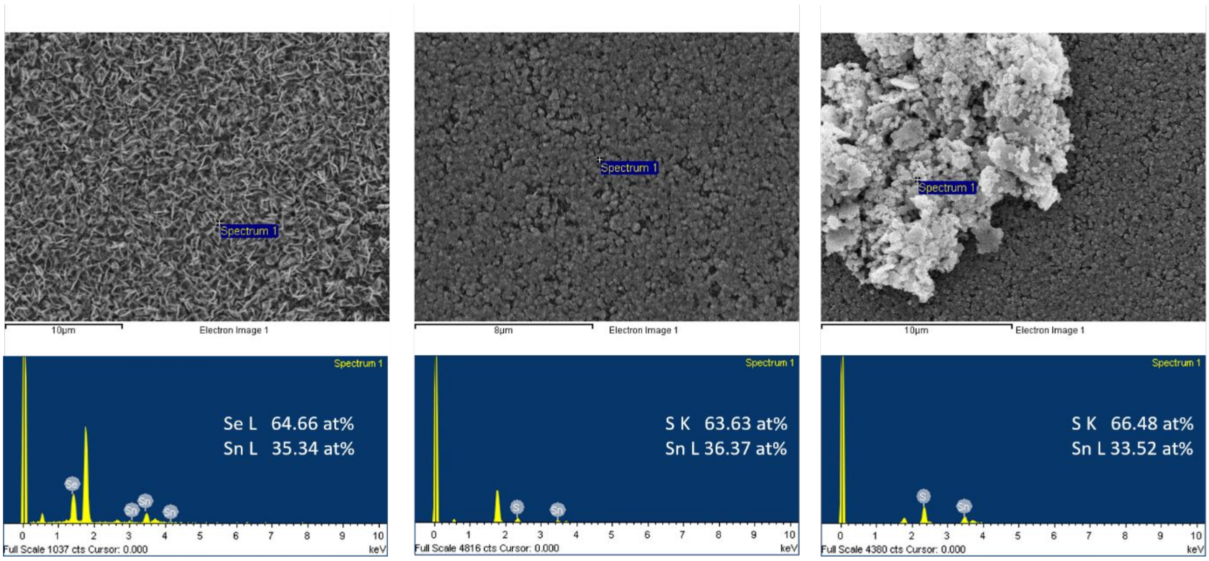

Figure S4. SEM micrographs (top) of tin chalcogenide thin films on Si/SiO_2 tiles. The yellow cross in each micrograph indicates the spot where EDS spectra were acquired. The EDS spectra are reported in the respective bottom panels.

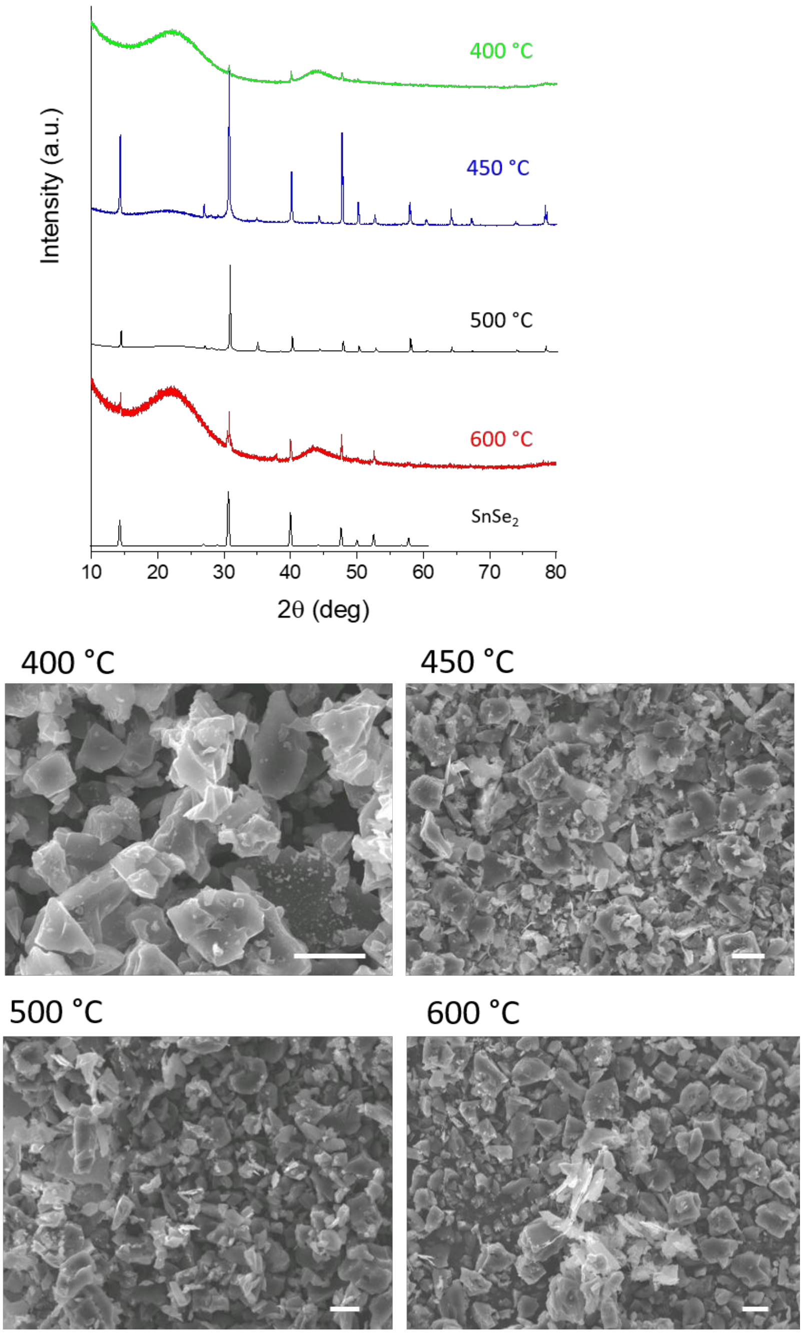

Figure S5. Powder XRD (top panel) and SEM of HC-$SnSe_2$ composites by plug flow CVD at different temperatures. The scale bars are 10 μm.

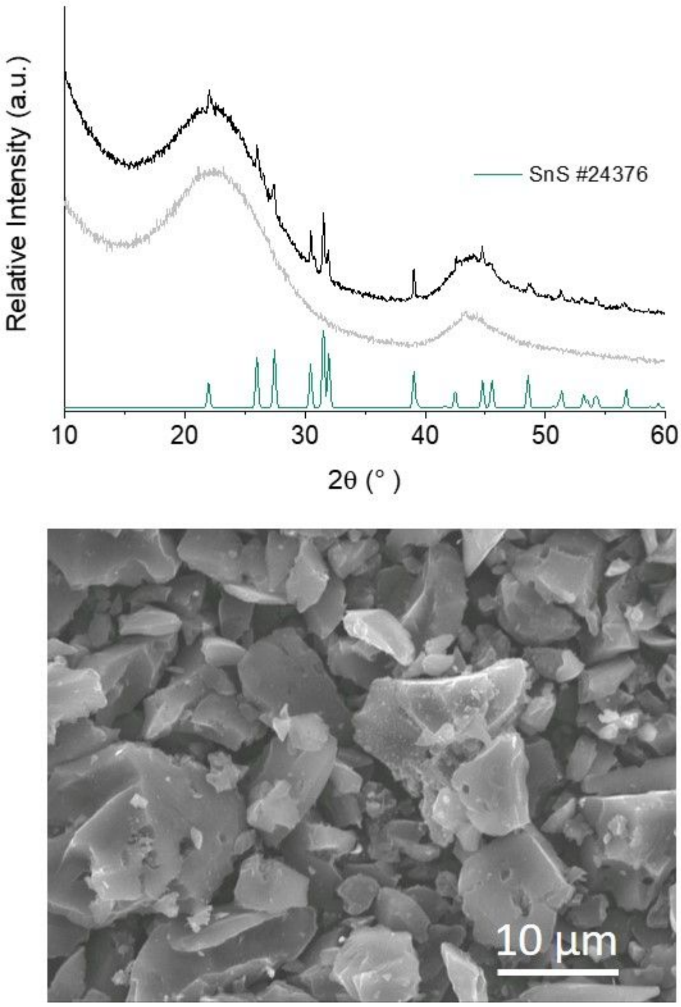

Figure S6. XRD (top panel) and SEM (bottom panel) of SnS_x deposited on HC powders by plug flow CVD.

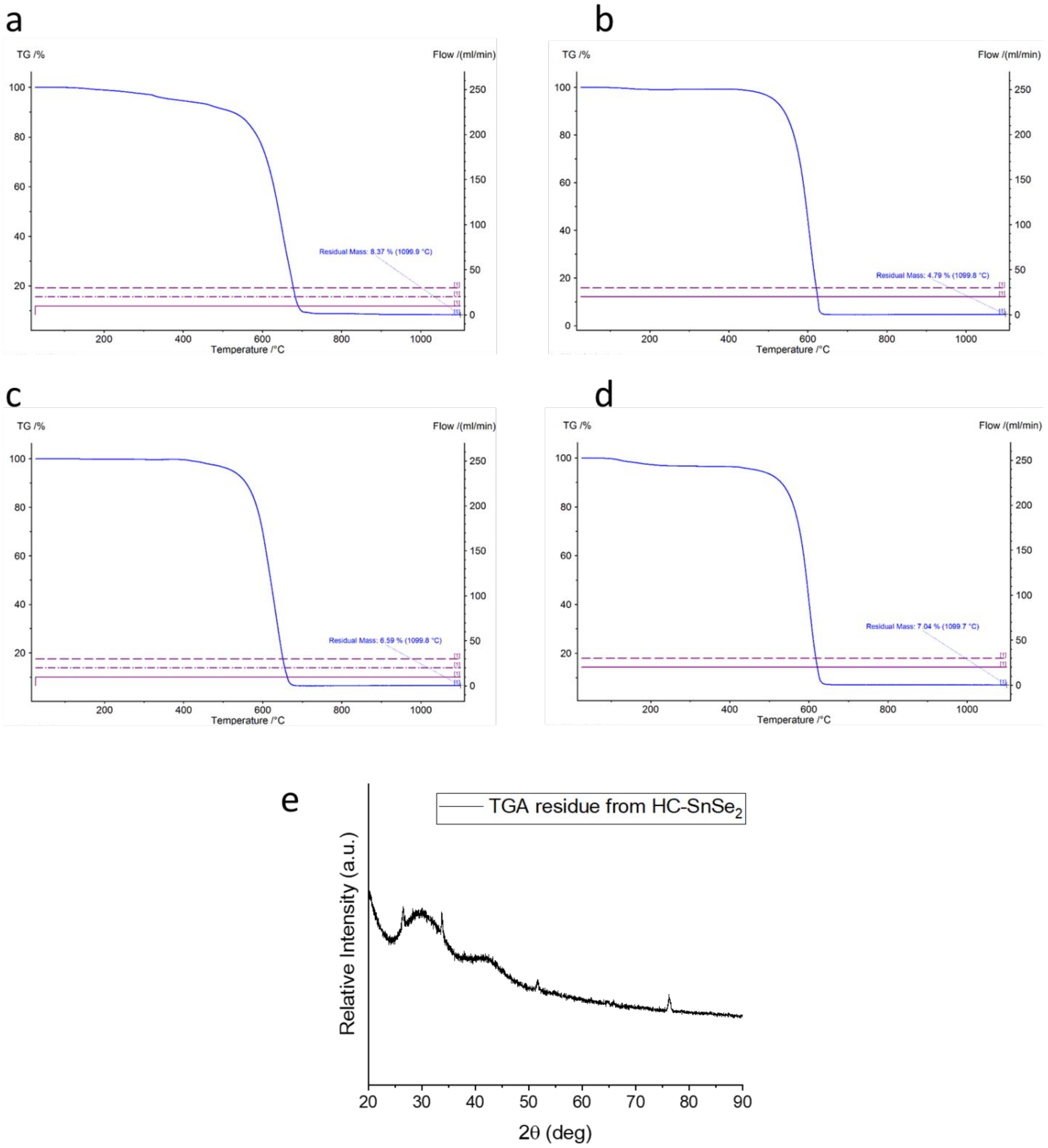

Figure S7. TGA (O_2 40% in Ar) of (a) HC-$SnSe_2$ composites synthesised by plug-flow CVD; (b) HC-$SnSe_2$ composites synthesised by FBCVD; (c) HC-$SnSe_2$ composites synthesised by FBCVD in a modified reactor configuration; (d) HC-SnS_x-$SnSe_2$ composites synthesised by FBCVD in a modified reactor configuration. (e) Representative powder XRD of the TGA residue: the sharp peaks are compatible with SnO_2 diffraction pattern. A similar pattern was observed for all samples.

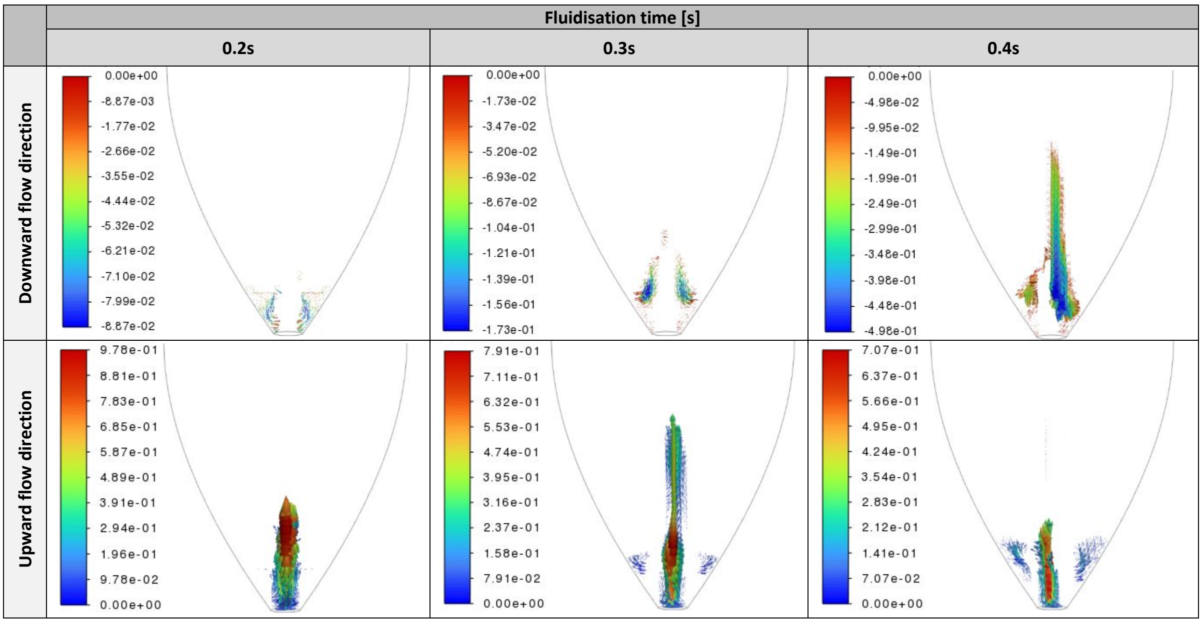

Figure S8. Close up of the specific directional y-velocities along the XY-plane for the 0.1 m/s inlet velocity at different times.

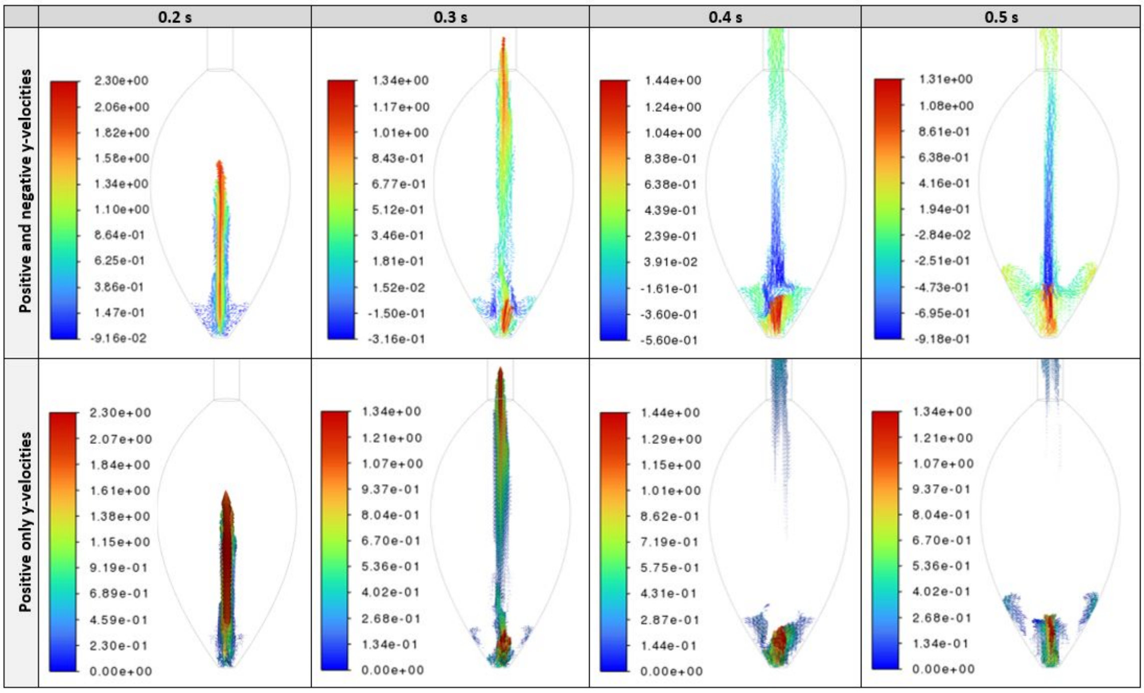

Figure S9. The positive and negative y-velocities (top) within the bulb along the XY-plane for the 0.4 m/s inlet velocity at varying fluidisation times; and the positive only y-velocities (bottom).

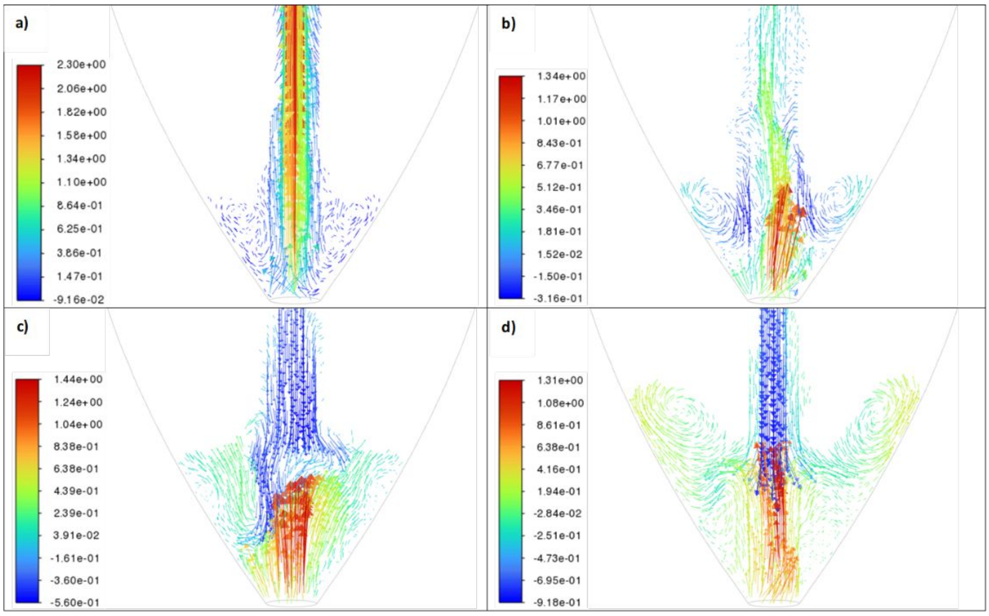

Figure S10. Close up of the positive and negative y-velocities along the XY-plane for the 0.4 m/s inlet velocity at a) 0.2s; b) 0.3s; c) 0.4s and d) 0.5s.

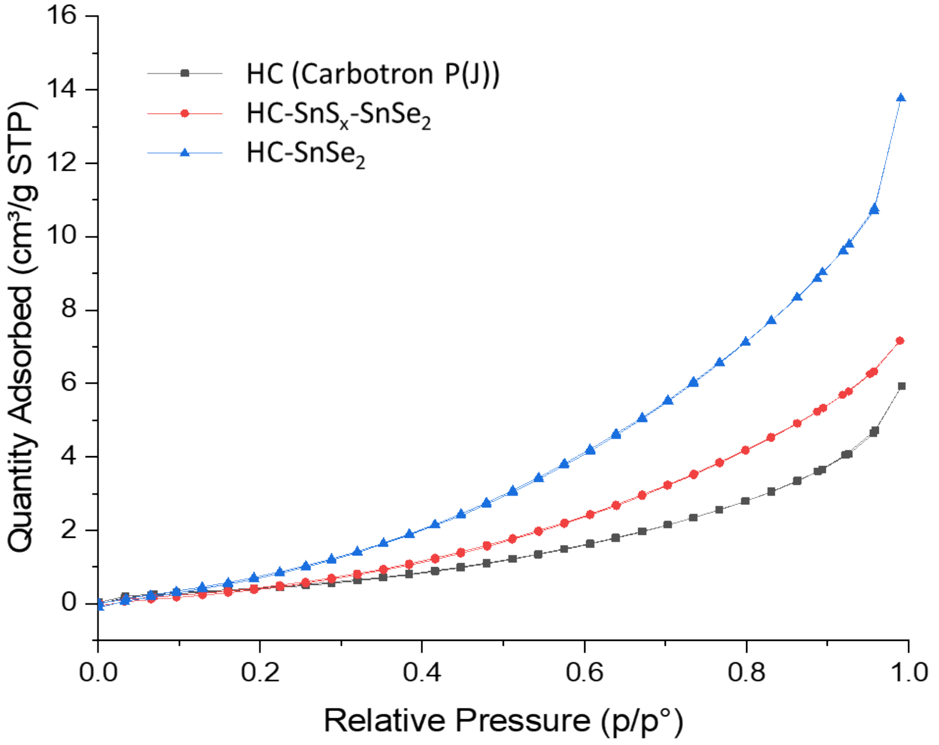

Figure S11. N_2 adsorption-desorption isotherms of pure HC and of the HC-$SnSe_2$ and HC-SnS_x-$SnSe_2$ samples obtained by FB-CVD.

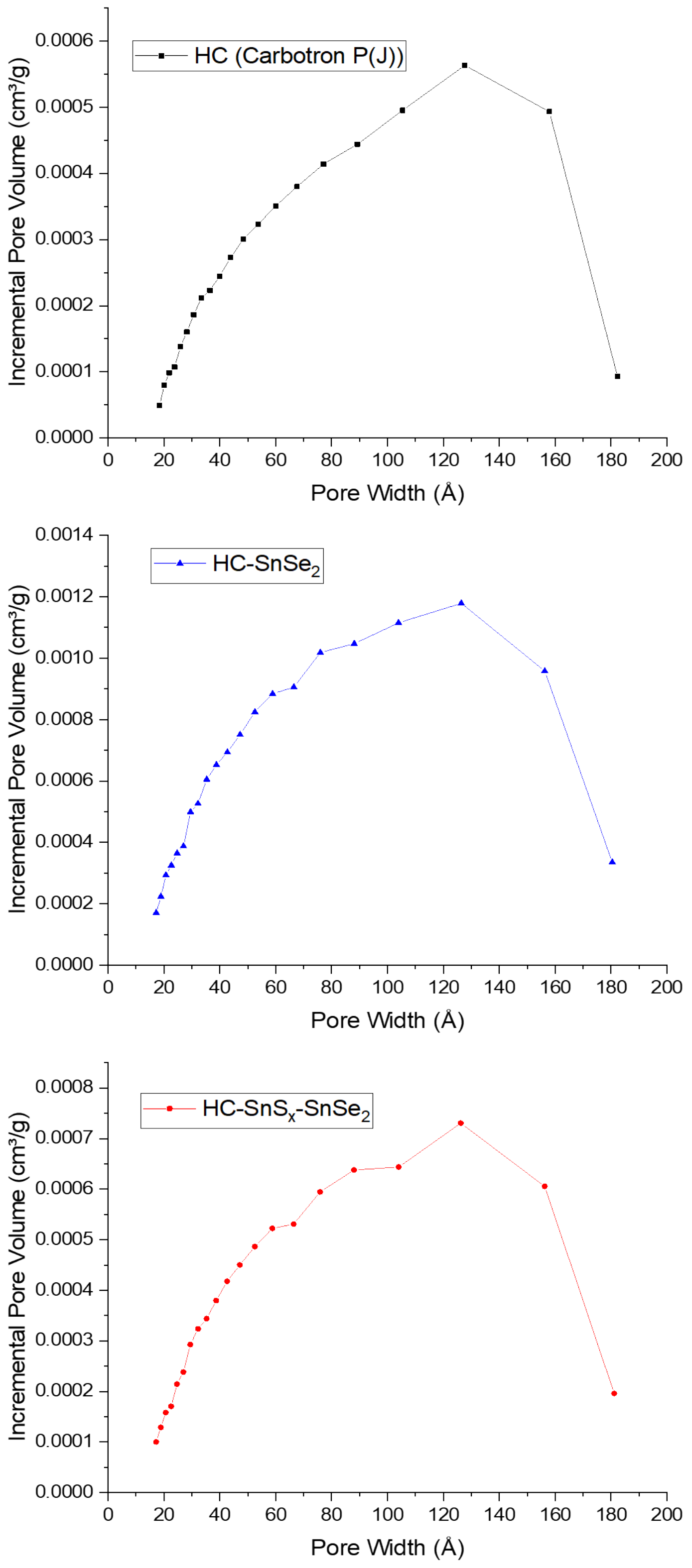

Figure S12. Pore size distribution of pure HC and of the HC-$SnSe_2$ and HC-SnS_x-$SnSe_2$ samples obtained by FB-CVD.
